# Supplementary material for: Spatially resolved photocatalytic active sites and quantum efficiency in a 2D semiconductor
Source: Nat Commun. 2025 Jul 26;16:6904. doi: 10.1038/s41467-025-62284-x (PMC12297338; doi:10.1038/s41467-025-62284-x)
Supplement: Supplementary file 1 — Supplementary Information [file 41467_2025_62284_MOESM1_ESM.docx]

Supplementary information of “Spatially Resolved Photocatalytic Active Sites and Quantum Efficiency in a 2D Semiconductor”

Olivier Henrotte^1,2^*, Seryio Saris^1^, Franz Gröbmeyer^1^, Christoph G. Gruber^1^, Ismail Bilgin^3^, Alexander Högele^3,4^, Naomi J. Halas^5,6,7,8^, Peter Nordlander^6,7,8^, Emiliano Cortés^1*^, and Alberto Naldoni^9*^

1 Nanoinstitute Munich, Fakultät für Physik, Ludwig-Maximilians-Universität München, 80539 München, Germany.

2 Regional Centre of Advanced Technologies and Materials, Czech Advanced Technology and Research Institute, Palacký University Olomouc, 779 00 Olomouc, Czech Republic.

3 Fakultät für Physik, Munich Quantum Center, and Center for NanoScience (CeNS), Ludwig-Maximilians-Universität München, Nanoinstitut München, Königinstraße 10, 80539 München, Germany.

4 Munich Center for Quantum Science and Technology (MCQST), Schellingstraße 4, 80799 München, Germany

5 Department of Chemistry, Rice University, Houston, TX, USA.

6 Department of Electrical and Computer Engineering, Rice University, Houston, TX, USA.

7 Department of Physics and Astronomy, Rice University, Houston, TX, USA.

8 Technical University of Munich (TUM) Institute for Advanced Study (IAS), Garching, Germany.

9 Department of Chemistry and NIS Centre, University of Turin, 10125 Turin, Italy

* Corresponding authors: [o.henrotte@lmu.de](mailto:o.henrotte@lmu.de) / [olivier.henrotte@upol.cz](mailto:olivier.henrotte@upol.cz) ; [Emiliano.cortes@lmu.de](mailto:Emiliano.cortes@lmu.de) ; [alberto.naldoni@unito.it](mailto:alberto.naldoni@unito.it)


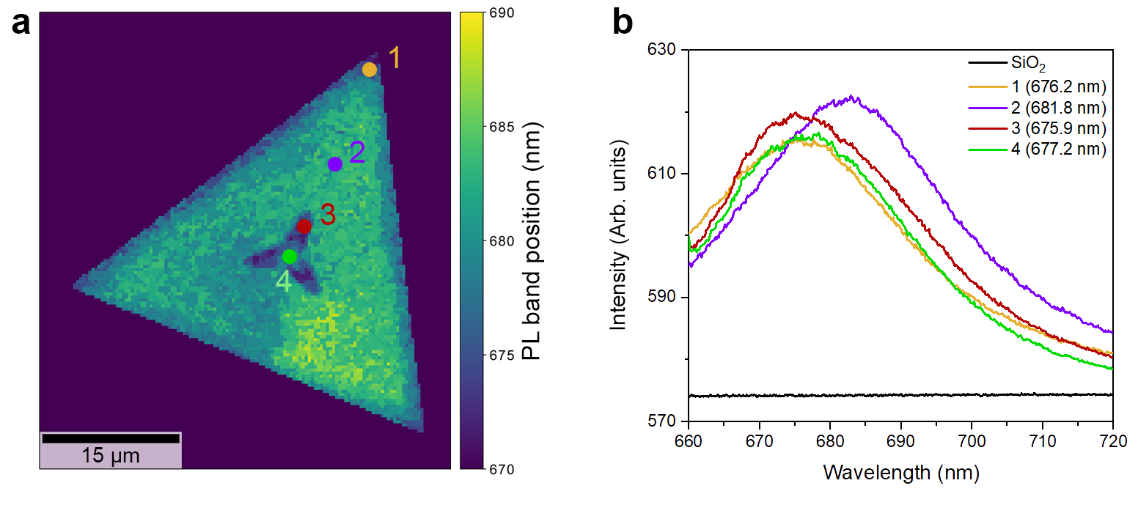


**Fig. S1:** **Photoluminescence microscopy of a single flake MoS_2_ with multilayer at the centre.** **a,** PL band position map of the MoS_2_ flake with multilayer at the centre. **b,** PL spectra for SiO_2_ (black) and position 1 to 4 (yellow, purple, brown and green, respectively) represented in (**a**) (circles of respective colors).


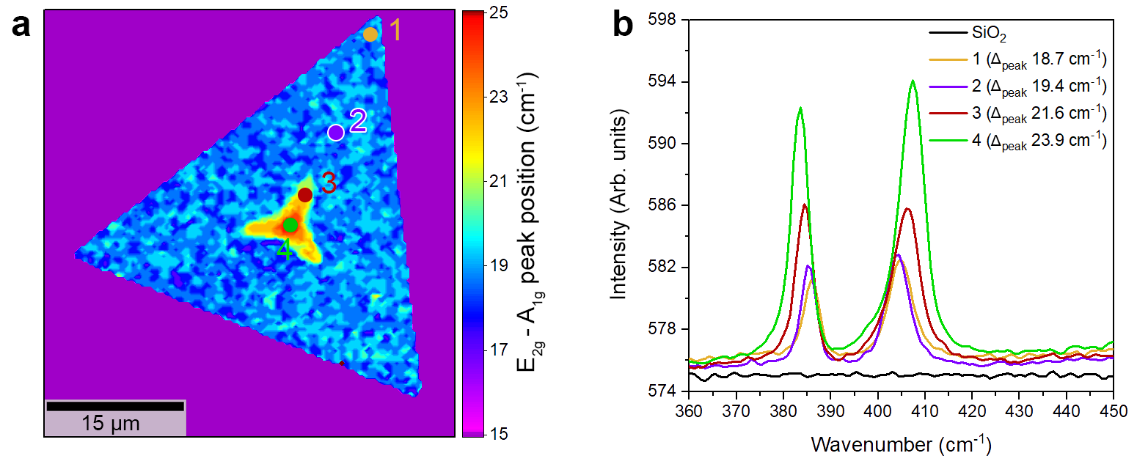


**Fig. S2:** **Raman microscopy of a single flake MoS_2_ with multilayer at the centre. a,** Raman peak difference (*Δ_peak_* = E_2g_-A_1g_) map of MoS_2_ flake with multilayer at the centre. **b,** Raman spectra for SiO_2_ (black) and position 1 to 4 (yellow, purple, brown, and green, respectively) represented in (**a**) (circles of respective colors).

**Supplementary Section 1: Details on the scanning photoelecotrchemical measurements.**

During the scanning photoelectrochemical microscopy (SPECM) measurements presented in Fig. 2, we conducted photo-oxidation and photoreduction maps to detect the photoproducts evolving from the MoS_2_ flake.

During the photo-oxidation map, we employed the ferrocene dimethanol (FcDM) to observe the reactivity of MoS_2_ under light excitation to extract charge carriers from the investigated surface. The following reactions occurred during the photo-oxidation map:

Photo-oxidation reaction at the MoS_2_ surface: $FcDM+h^{+}\to{FcDM}^{+}$ Eq. S1

Photo-reduction reaction at the MoS_2_ surface: ${FcDM}^{+}+e^{-}\to FcDM$ Eq. S2

Electrochemical reduction at the probe: ${FcDM}^{+}+e^{-}\to FcDM$ Eq. S3

The electrochemical probe was biased at a potential corresponding to the diffusion limiting current of the species of interest (-0.1 V vs Ag/AgCl, KCl 3.4M), corresponding to FcDM^+^ reduction in this case. Consequently, the measured photoactivity (ΔI = I_T,Light_ – I_T,Dark_) reflects the number of FcDM molecules effectively oxidized by the MoS_2_ and diffusing to the probe. Due to the counter reaction occurring at the MoS_2_ flake and the hindrance caused by the probe, the apparent activity of the basal plane (Fig. S3a) and the corner (Fig. S3b) are influenced by the surrounding species produced away from the excitation spot, since in every position in the solution the sum of FcDM and FcDM^+^ molecules remain constant. Therefore, the measured |ΔI| is decreased due to photoreduction occurring around the excitation spot and the diffusion of species being impeded by the probe.


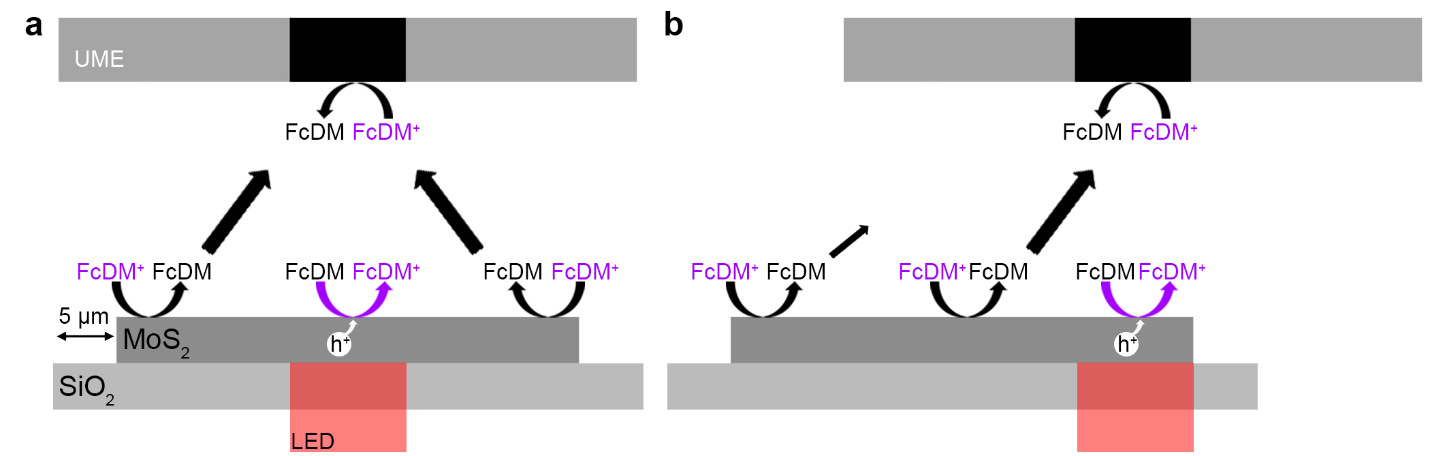


**Fig. S3: Influence of the electrochemical probe position on the ferrocene dimethanol diffusion.** Schemes describing the situation where the probe is detecting the species at the centre (**a**) or at the edge (**b**) of the MoS_2_ flake during the photo-oxidation map. The probe size, the light illumination spot, and the distance between the probe and the investigated material correspond to the conditions of the photo-oxidation map presented in Fig. 2e, according to the scalebar of 5 µm. UME: ultra-microelectrode.

For the photoreduction experiments, water electroreduction is the most studied catalytic reaction on MoS_2_. As such, we investigated the water reduction under photocatalytic conditions on bare MoS_2_ monolayer, which is usually performed with hybrid materials.^1,2^ We performed the measurements in the presence of a hole scavenger to enhance the photogenerated electron lifetime, and remove the contribution of the oxidized species on the measured current at the probe. The following reactions occurred during photoreduction experiments:^3^

Photo-oxidation reaction at the MoS_2_ surface: ${SO}_{3}^{2-}+{2h}^{+}+H_{2}O\to{SO}_{4}^{2-}+2H^{+}$ Eq. S4

Photo-reduction reaction at the MoS_2_ surface: $2H^{+}+2e^{-}\to H_{2}$ Eq. S5

Electrochemical oxidation at the probe: $H_{2}\to2H^{+}+2e^{-}$ Eq. S6

From the ΔI (~0.5 pA), we can estimate the concentration of H_2_ collected at the probe:

$I_{T}=4nFCDr_{T}$ Eq. S7

where n is the number of electrons involved (2); F, the faraday constant (96485.33 s.A/mol); C, the concentration in Red/Ox species; and D, the diffusion coefficient of the Red/Ox species (4.5x10^-5^ cm²/s for H_2_).^4^ Thus, a concentration of 28.8 nM of H_2_ can be calculated from the photoactivity recorded at the probe, revealing a poor reactivity if we consider the incident light of 7.64 W.cm^-2^. For comparison, the FcDM^+^ estimated concentration detected at the probe (corresponding to -7 pA) is 4.6 µM. Consequently, the H_2_ generation is at least 80 times less effective than the FcDM oxidation despite an incident illumination 20 times higher and a probe-distance 4 times lower.

In the case where the highest photoactivity was observed at the basal plane in Fig. 2f, two possible scenarios can explain our results: (1) H_2_ is produced directly at the light excitation spot and subsequently detected by the probe (Fig. S4a), or (2) H_2_ is produced at reactive sites and diffuses to the probe due to mass transport hindrance caused by the probe itself (Fig. S4b). Considering that the reactive sites of MoS_2_ are well-known to be defect sites, as evidenced by PL mapping at the edges of the MoS_2_ (Fig. 1a), the second scenario is the most plausible explanation for our observations.


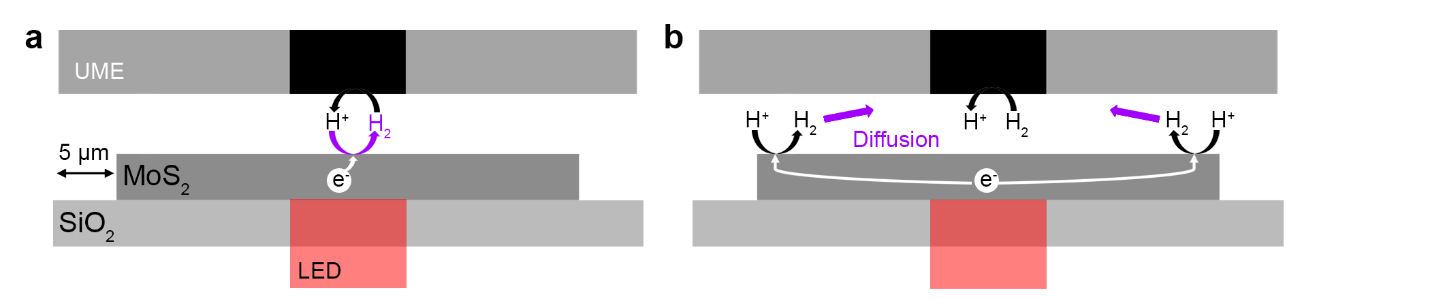


**Fig. S4:** **Proposed mechanisms of photoreduction detection at the basal plane according to electron pathways.** Schemes describing the proposed scenarios, where the probe detects the species generated directly at the basal plane due to the photoreduction process occurring locally at the light excitation (**a**), or the probe detects the species generated at the edge of the MoS_2_ flake due to the electron migration to the reactive sites and the subsequent hindered diffusion of the species related to the probe size (**b**). The probe size, the light illumination spot, and the distance between the probe and the investigated material represent the conditions during to the photoreduction map presented in Fig. 2f, according to the scalebar of 5 µm. UME: ultra-microelectrode.

To understand the effect of the probe hindrance on the generation of molecules according to the probe’s position, we simulated the diffusion of molecules from the surface considering their production at the light position (Fig. S5), as in Fig. S4a. We observe similar diffusion profile independently of the position in the flake as the irradiated area remain the same, which does not correspond to the experimental results obtained in Fig. 2f.


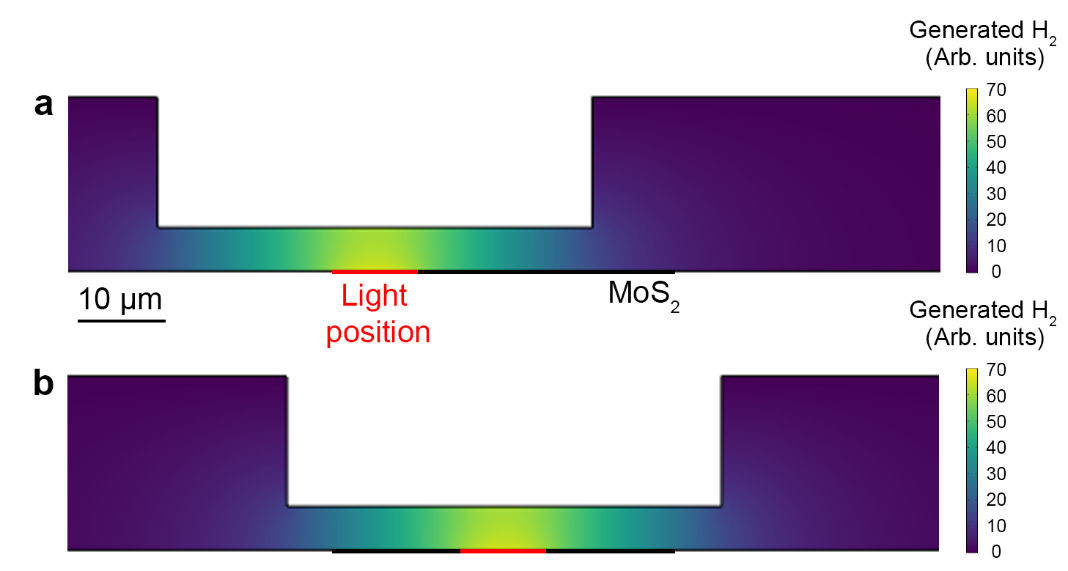


**Fig. S5: Simulated hydrogen generation at the light position on MoS_2_ according to the aligned probe position.** Simulation model results obtained for the production of H_2_ at the light excitation spot in presence of the probe aligned with the light positioned at the corner (**a**) or at the centre (**b**) of the MoS_2_ flake. The probe size, the light illumination spot, and the distance between the probe and the investigated material represent the conditions during to the photoreduction map presented in Fig. 2f, according to the scalebar of 10 µm.


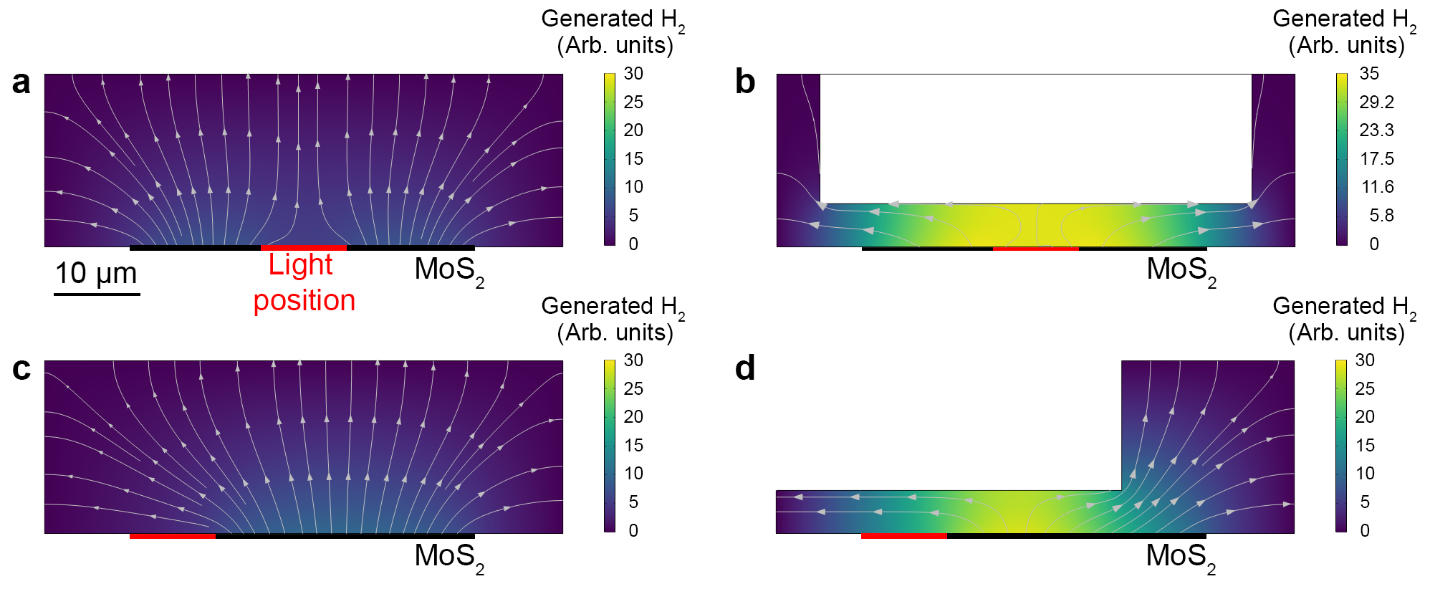


**Fig. S6: Simulated hydrogen generation away from the light position on MoS_2_ according to the aligned probe position.** Simulation model results obtained for the production of H_2_ away from the light excitation spot without the probe (**a,c**) or with the probe aligned with the light (**b,d**) positioned at the centre (**a,b**) or at the corner (**c,d**) of the MoS_2_ flake. The probe size, the light illumination spot, and the distance between the probe and the investigated material represent the conditions during to the photoreduction map presented in Fig. 2f, according to the scalebar of 10 µm.

To understand the hindering effect from the probe on the measurements, a dimensionless parameter has been previously introduced as L,^5^ corresponding to the ratio between the probe-substrate distance (Z) and the active part radius (r_T_). For L ≥ 10, no hindering effect is expected. For the measurements in Fig. 2f, L = 1, which suggests a significant hindrance from the system on the diffusion, forcing the molecules to diffuse from the MoS_2_ to the probe. To highlight the hindrance of the probe on the generated species, we simulated with the same model the scenario represented in Fig. S4b in absence (Fig. S6a,c) and presence of the electrochemical probe (Fig S6b,d) at the centre and the corner of the MoS_2_ flake. This reveals i) the significant hindrance of the probe on the diffusion of H_2_ in solution, and ii) a similar trend than the one observed in Fig 2f with H_2_ detected at the probe in higher quantity at the centre than the corner of the MoS_2_ flake (Fig. S6b,d). Furthermore, we believe that the detection of H_2_ in this system was possible due to the probe hindrance, as bare MoS_2_ exhibits significantly low photogeneration of H_2_.

To avoid this contribution in the measurements performed in Fig. 3 and 4, a nanoprobe (r_T_ = 100 nm) at a probe-substrate distance of 5 µm was used, corresponding to L = 50. Moreover, Z was chosen to keep a distance larger than 2 r_ins_ to completely remove any suspicions coming from the hindering effect of the nanoprobe during those measurements.


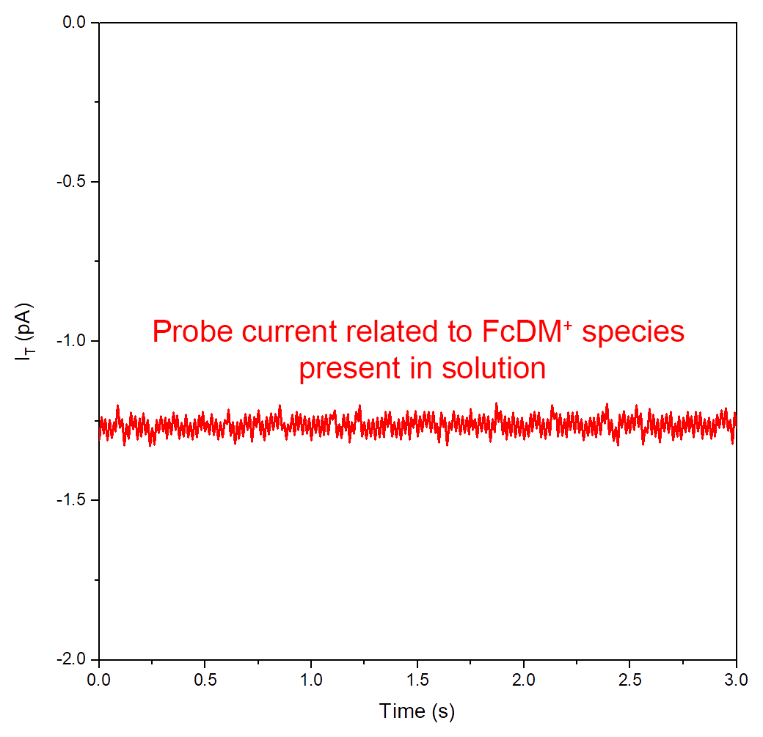


**Fig. S7: Detection of the ferrocenium dimethanol traces present in solution.** Chronoamperometry of the probe current under dark conditions in presence of 1 mM of FcDM in KCl 0.1 M at E_Tip_ = -0.1 V (vs Ag/AgCl in KCl 3.4 M).

**Supplementary Section 2: Influence of the FcDM^+^ species on the observed photoactivity.**

To better illustrate the effect of the FcDM⁺ species on system limitations, we performed aligned-unaligned excitation-detection measurements on freshly prepared and one-year-old 1 mM FcDM solutions. Due to the equilibrium between FcDM and FcDM⁺ in water, the aging process resulted in a shift toward a lower FcDM concentration (and a correspondingly higher FcDM⁺ concentration) in the one-year-old solution (Fig. S8).


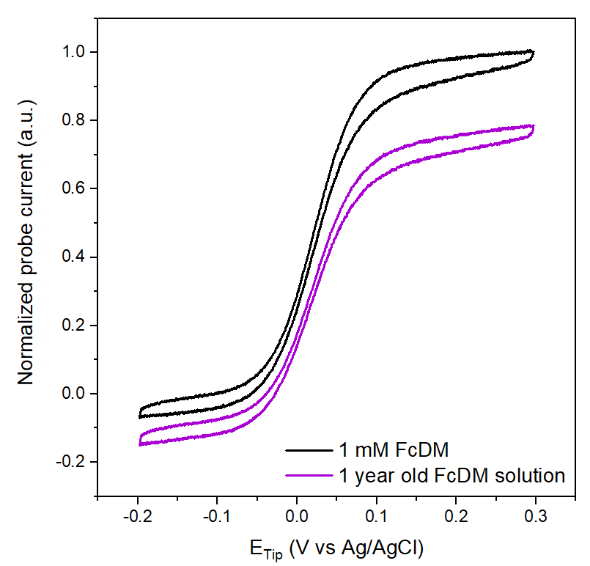


**Fig. S8:** **Comparison of electrochemical signal according to the aging of the solution.** Cyclic voltammetry of 1 mM FcDM solution freshly prepared (dark) and 1-year-old (purple) representing the normalized probe current according to the probe current obtained at the oxidation peak for the 1 mM FcDM fresh solution.

This decrease in the reduced species (FcDM) and corresponding increase in the oxidized species (FcDM⁺) influence the extraction efficiency of photogenerated charge carriers during the measurements. The relative signal intensity provides direct information on species concentration, as only the species concentration varies (Eq. S7). Considering a normalized probe current of 1 for the fresh 1 mM FcDM solution, the FcDM⁺ species exhibited a normalized intensity of 0.06. In contrast, the one-year-old solution yielded normalized intensities of 0.78 and 0.15 for FcDM and FcDM⁺, respectively.

As in Fig. 3, we conducted aligned-unaligned excitation-detection measurements for both solutions and compared the results (Fig. S9). Interestingly, the aligned measurements exhibited similar photoactivities. However, the one-year-old solution displayed a slightly more negative photoactivity for both C and A transitions. The most significant difference emerged in measurements taken away from the illumination position (as indicated in Fig. S9a). While the fresh FcDM solution exhibited a signal comparable to that shown in Fig. 3, no detectable signal was observed for the one-year-old FcDM solution (Fig. S9b,c). This can be attributed to the changes in the availability of FcDM/FcDM⁺ species: the concentration of FcDM molecules available for hole extraction decreased by approximately 20%, whereas the availability of FcDM⁺ for electron extraction increased by nearly 200%. Consequently, the local concentration of FcDM⁺ became less limiting compared to the fresh FcDM solution.


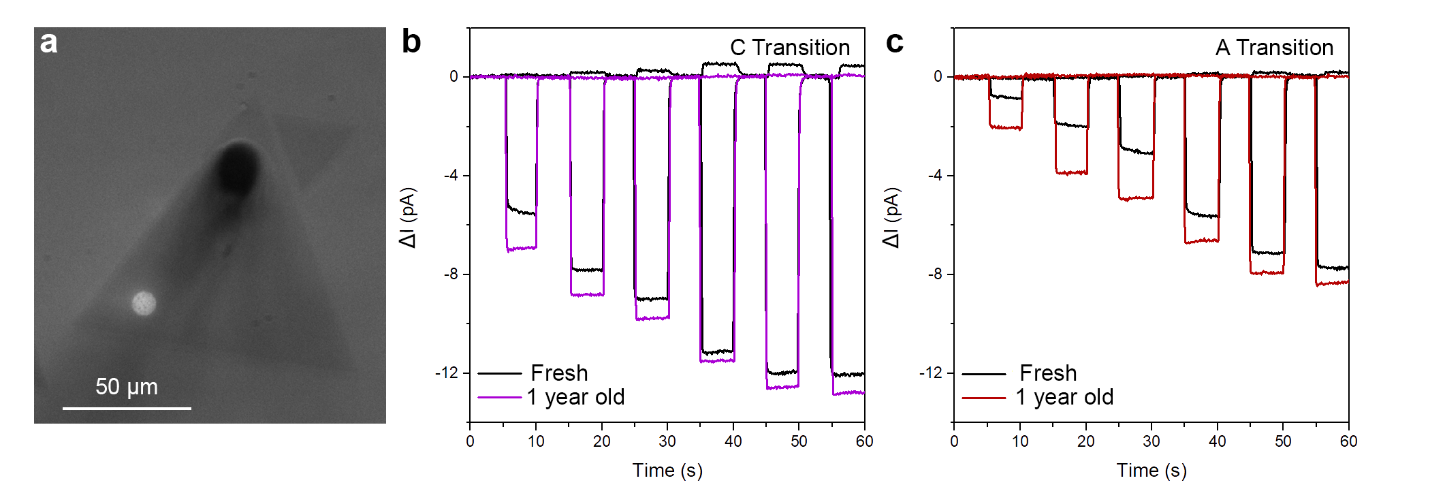


**Fig. S9: Influence of the solution on the aligned-unaligned excitation-detection experiments in monolayer MoS_2_. a,** Optical image showing the monolayer (ML-) MoS_2_ flake, the position of the light excitation spot, and the ultramicroelectrode (UME) position corresponding to the unaligned excitation-detection measurements. For the aligned excitation-detection measurements, the UME was positioned at the light excitation spot. (**b,c**) Photoactivity measurements under chopped light excitation (5 s; increased light power at every pulse) for the aligned (negative ΔI) and unaligned (positive ΔI) measurements for the C transition (**b**) and the A transition (**c**) performed in presence of fresh 1 mM FcDM (black curves) and one-year-old 1 mM FcDM (colored curves) solutions. Conditions: r_T_ = 0.1 µm, RG = 25, and Z = 5 µm for (**b,c**). The initial solutions were prepared with 1 mM FcDM and 0.1 M KCl.

To further investigate the regions where reduction products formed on the MoS₂ flake immersed in the one-year-old FcDM solution, we performed unaligned excitation-detection measurements at different locations along the flake (Fig. S10). We selected four positions for probing photoproducts: (1) far from the illumination site (~75 µm), (2) mid-distance (~30 µm), (3) near the illumination site (~10 µm), and (4) directly adjacent to the illuminated region (~1–2 µm) (Fig. S10a). Notably, photoactivity associated with reduced products peaked at position 3 for the C transition (Fig. S10b) and at position 4 for the A transition (Fig. S10c). A sharp increase in signal intensity was observed immediately upon illumination, followed by a decrease, suggesting that reduction occurs instantaneously while oxidized products diffuse from the excitation site toward the probe (Fig. S10b). This effect was particularly pronounced for the C transition at position 4, where the high diffusion of FcDM⁺ resulted in a decrease in photoactivity, ultimately yielding negative ΔI values. In contrast, the species generated under excitation at the A transition exhibited little to no diffusion. This behavior may be attributed to differences in carrier dynamics, as A-excitons correspond to more localized and tightly bound carriers compared to those associated with the C transition. Furthermore, the absence of photoactivity at position 1 indicates that electrons no longer reached this region. We hypothesize that the combination of the excitation spot size and the decrease in FcDM concentration acted as limiting factors in the one-year-old FcDM solution. This highlights the critical influence of electrolyte composition (availability of species to be reduced and oxidized) and light excitation position on the photoreaction dynamics occurring at the MoS_2_ platform.


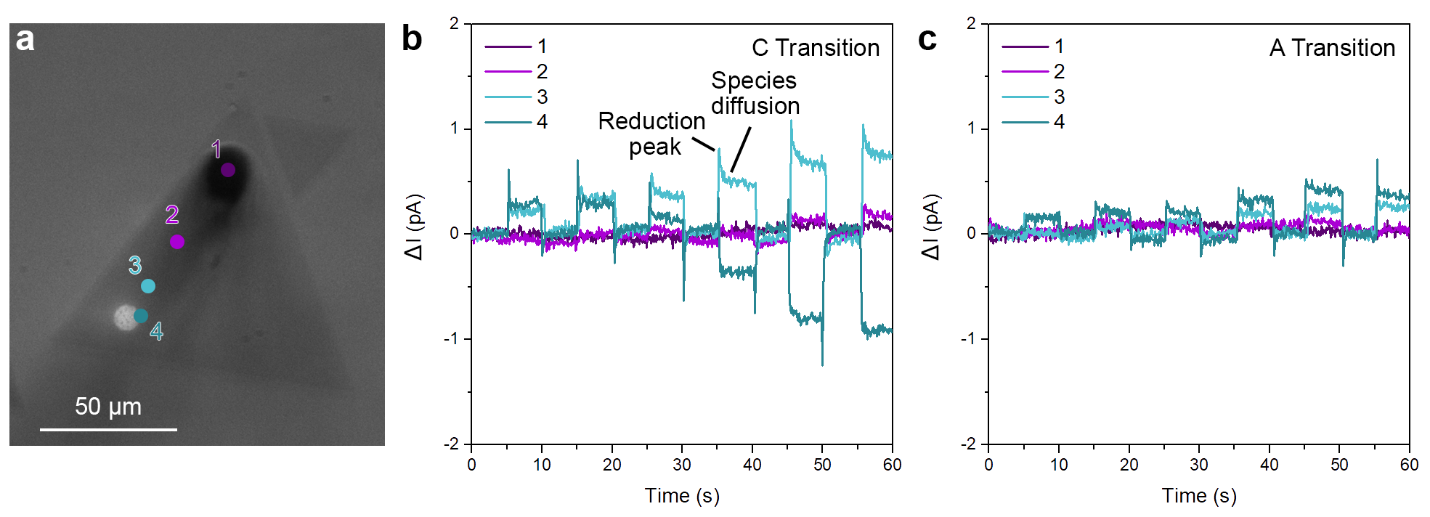


**Fig. S10: Influence of the probe position during aligned-unaligned excitation-detection experiments in monolayer MoS_2_. a,** Optical image showing the monolayer (ML-) MoS_2_ flake, the position of the light excitation spot, and the ultramicroelectrode (UME) positions corresponding to the unaligned excitation-detection measurements performed at the different positions indicated on the image: (1) far from the illumination site (~75 µm), (2) mid-distance (~30 µm), (3) near the illumination site (~10 µm), and (4) directly adjacent to the illuminated region (~1–2 µm). (**b,c**) Photoactivity measurements under chopped light excitation (5 s; increased light power at every pulse) at the positions 1 to 4 (dark purple, purple, teal, and dark teal, respectively) indicated in (**a**) (circles of respective colors) for the C transition (**b**) and the A transition (**c**) performed in presence of one-year-old 1 mM FcDM solutions. Conditions: r_T_ = 0.1 µm, RG = 25, and Z = 5 µm for (**b,c**). The initial solution was prepared with 1 mM FcDM and 0.1 M KCl.


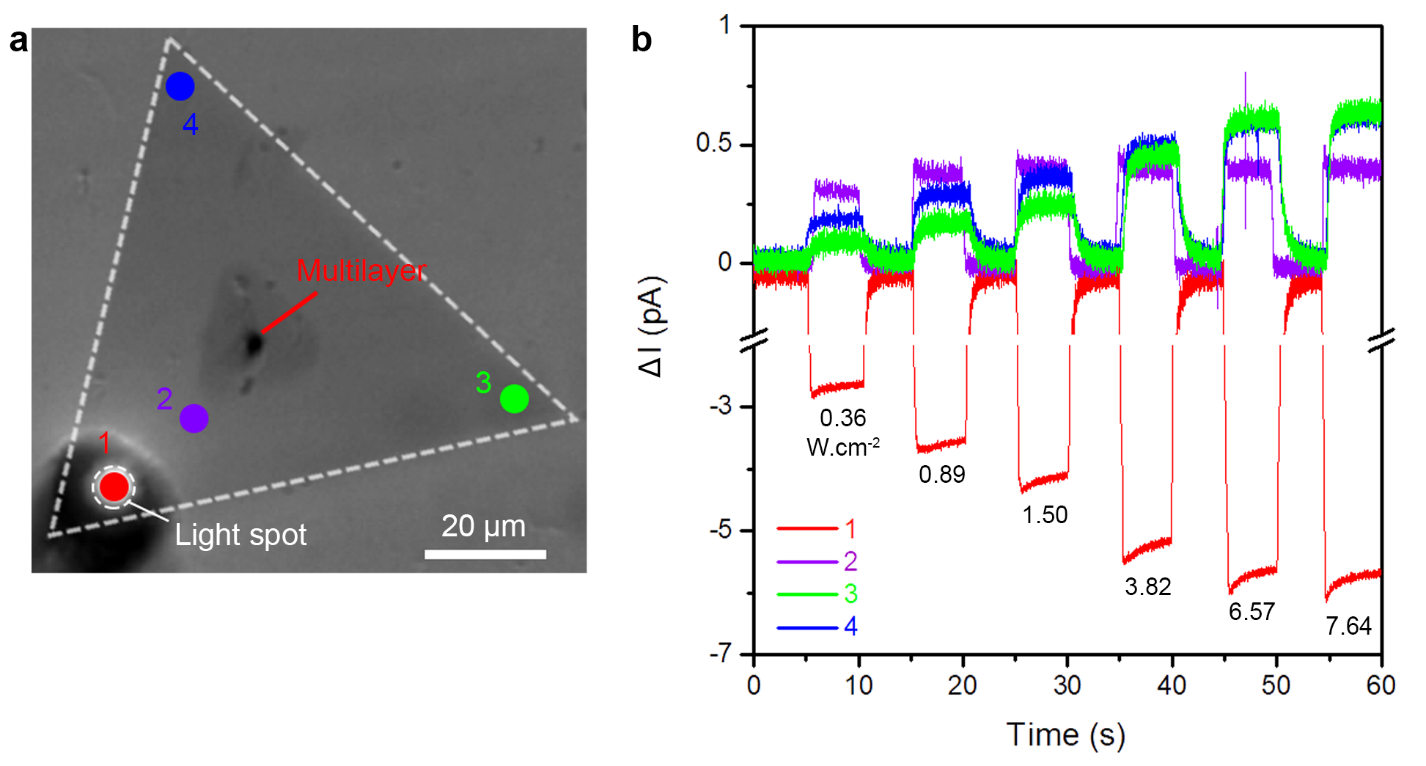


**Fig. S11:** **Aligned-unaligned excitation-detection experiments on a large flake of MoS_2_. a,** Optical image of the large ML-MoS_2_ (dashed white triangle) flake showing the position of the light excitation spot (white dashed circle) and the different probe position for (1, red circle) aligned and (2, 3, and 4; purple, green and blue circles, respectively) unaligned measurements. **b,** Photoactivity measured at the different positions represented in (**a**), while the ML-MoS_2_ was excited under chopped light (5 s; increased light power at every pulse with λ = 595 nm) at the light spot position indicated in (**a**). Conditions: r_T_ = 0.1 µm, RG = 25, and Z = 5 µm for (**b**). The experiments were performed in aqueous solutions containing 1 mM FcDM + KCl 0.1 M.


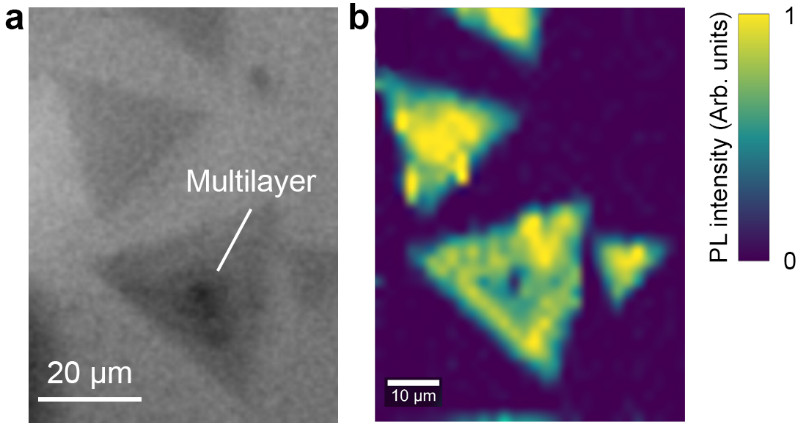


**Fig. S12:** **Photoluminescence microscopy of the small studied MoS_2_ flakes. a**, Optical image of Flake 1 and Flake 2 from Fig. 4, highlighting the presence of a multilayer at the centre of Flake 1. **b**, Normalized PL peak intensity map of the MoS_2_ flake in (**a**).

**Supplementary Section 3: Effect of the MoS_2_ flake morphology on the quantum efficiency.**

To investigate the influence of MoS_2_ flake morphology on its photocatalytic efficiency, we selected a monolayer flake with a small multilayer region at its centre, similar to the one depicted in Fig. 1. Aligned excitation-detection measurements were performed to ensure the reliability of our results and to examine the impact of the multilayer region on quantum efficiency (QE). As shown in Fig. S13, measurements were conducted at three distinct locations: the flake's corner (purple circle), the multilayer-containing centre (dark red circle), and the basal plane, away from the centre and free of multilayer regions (grey circle).


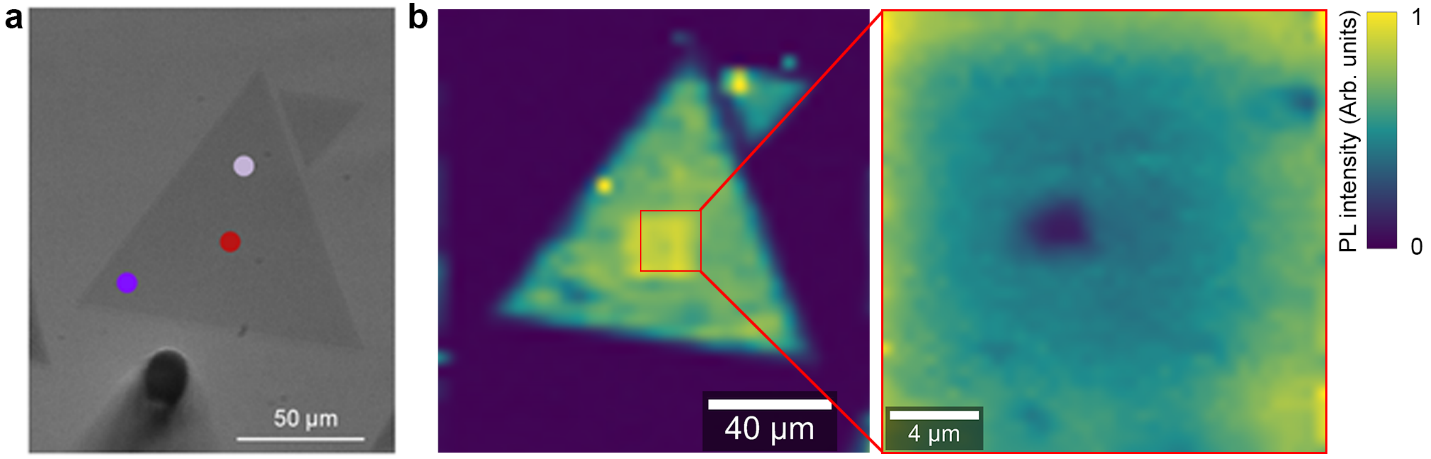


**Fig. S13:** **Photoluminescence microscopy of the large studied MoS_2_ flakes. a**, Optical image corresponding to the investigated monolayer MoS_2_ flake with the different positions where measurements were performed: purple for corner, dark red for basal at the centre with the presence of multilayer (namely, basal (multi)), and grey for basal away from the centre to avoid any multilayer (namely, basal (mono)). **b**, Normalized PL peak intensity map of the MoS_2_ flake in (**a**). The inset shows the high-resolution PL peak intensity map of the region containing multilayers.

For each position, we evaluated the external quantum efficiency (EQE) and internal quantum efficiency (IQE) for the C and A transitions under varying photon fluxes (Fig. S14). Notably, at the basal plane, the QE exhibited a substantial difference between the C and A transitions, depending on the presence or absence of the multilayer. Specifically, the QE for the A transition was significantly reduced in the presence of a multilayer compared to a purely monolayer region (Fig. S14a,b). In contrast, the QEs for the C transition remained largely unchanged. These findings suggest that the multilayer region impedes charge carrier extraction from bound excitons at the MoS₂-liquid interface, thereby decreasing the overall QE of the system.

Interestingly, under high photon flux, this difference became less pronounced, as spatial constraints imposed by the MoS_2_ flake dimensions and the availability of charge-extracting molecules in solution remained fixed (Fig. S14c,d). The increased photon flux led to a greater number of charge generation events, which enhanced charge carrier extraction efficiency until the system reached its inherent limitations.


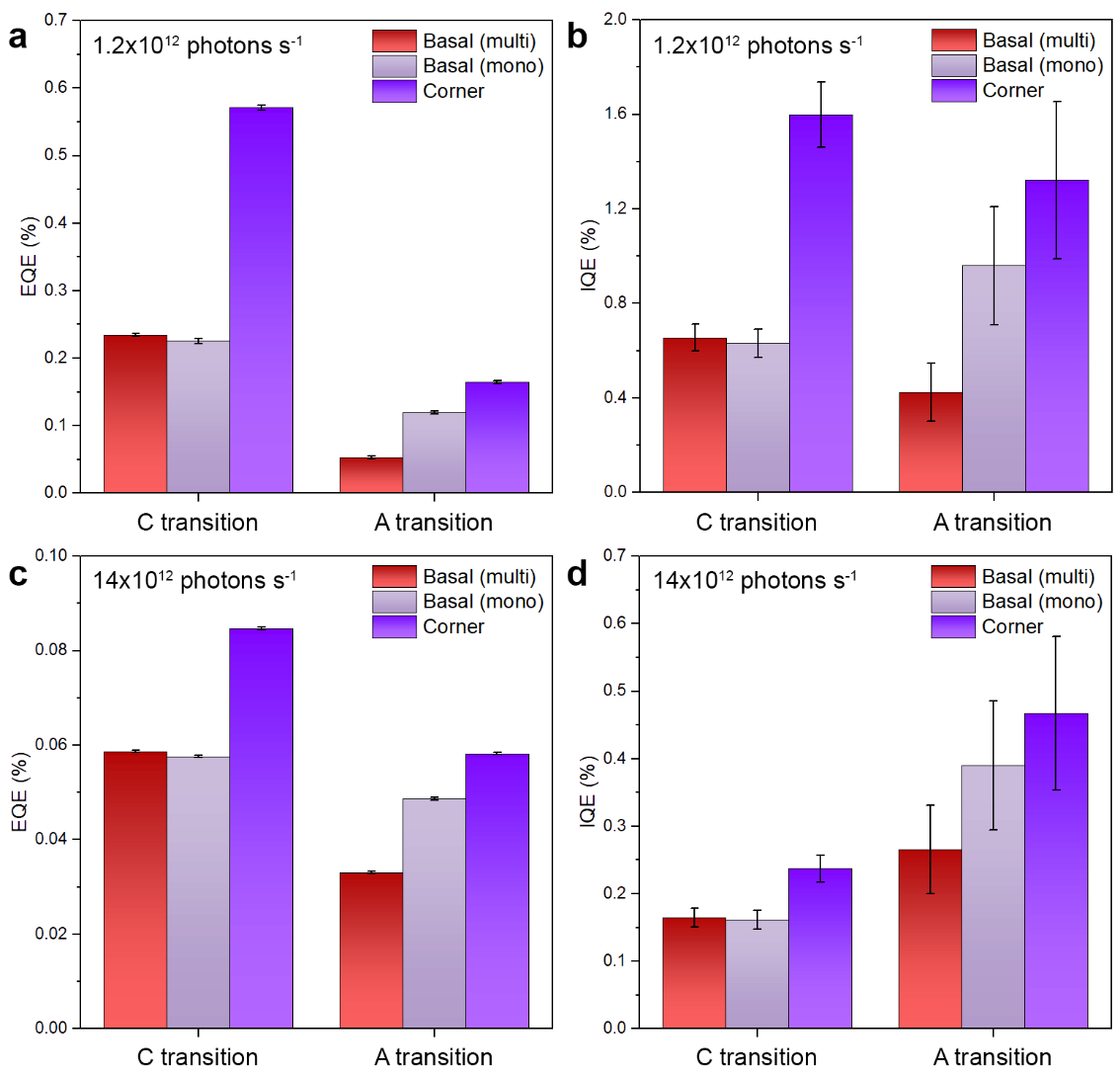


**Fig. S14:** **Quantum efficiency evolution according to the position on MoS_2_.** (**a,c**) External and (**b,d**) internal quantum efficiency at different positions (basal (multi): dark red; basal (mono): grey; corner: purple) inside the ML-MoS_2_ (Fig. S13a) according to the excited transition at (**a,b**) ~1.2$\times$10^12^ and (**c,d**) ~14$\times$10^12^ photons s^-1^ (C transition at 455 nm; A transition at 660 nm). The error bars represent the standard deviation.

To assess the role of flake size in QE measurements, we studied MoS_2_ flakes of varying sizes relative to the excitation beam (approximately 80 µm²). The selected flakes ranged from 0.5 to 70 times the beam area (Fig. S15).


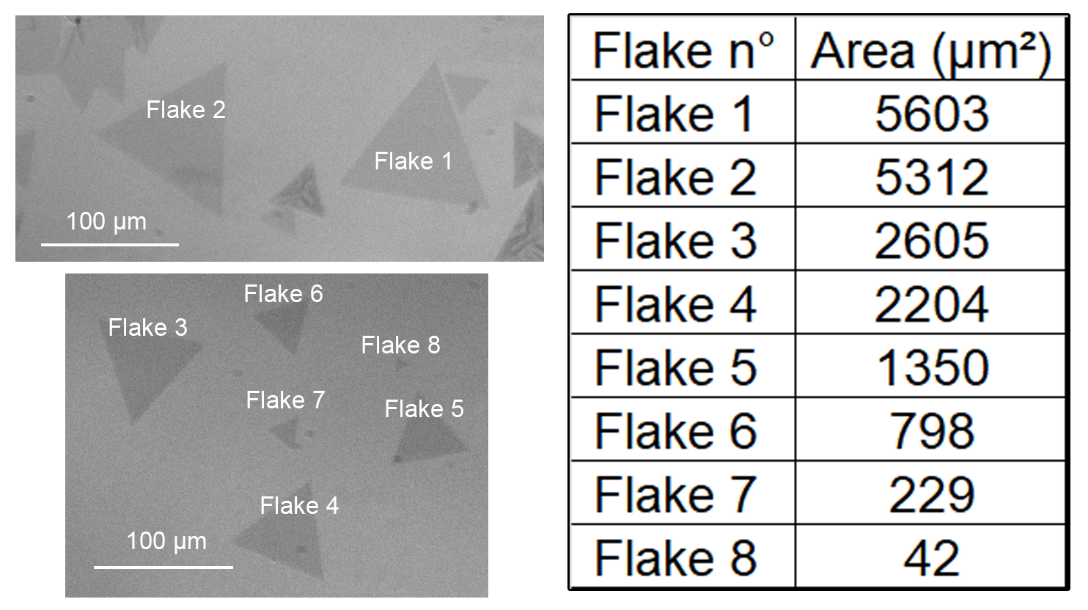


**Fig. S15: Population distribution of the investigated MoS_2_ flakes.** On the left, optical images of the different flakes investigated for comparing the flake size. On the right, table containing the flake number and the corresponding area measured with ImageJ.

Measurements of EQE (Fig. S16a,c) and IQE (Fig. S16b,d) were conducted at different photon fluxes (~1.2×10¹² s⁻¹, Fig. S16a,b; ~14×10¹² s⁻¹, Fig. S16c,d) at the centre of each flake, which were numbered according to their size, from the largest (Flake 1) to the smallest (Flake 8). At low photon flux, EQE and IQE remained consistent for Flakes 1 to 6 but dropped significantly for Flakes 7 and 8. This reduction was less pronounced for Flake 7 under A transition excitation. These results suggest that QE is governed by multiple factors, including flake size, excitation spot size, and the availability of charge-extracting species in solution. For flakes with an area close to the light spot size, photocarrier saturation occurred even at the lowest studied intensities. Conversely, for flakes much larger than the excitation beam (10 times or more), the maximum QE was limited by charge carrier interaction with the solution rather than by spatial confinement effects. Consequently, similar QE values were observed for all large flakes (Flakes 1 to 6).

Under higher photon flux (Fig. S16c,d), the overall trend remained unchanged, except for Flakes 5 and 6 under C transition excitation. The observed reduction in QE for these flakes suggests a transition in the limiting factor from charge carrier availability at the MoS_2_-liquid interface to the MoS_2_ surface area available for charge extraction.

Ultimately, our findings highlight the complexity of understanding photocatalytic behavior, even in a simple system like MoS_2_. Our results underscore the importance of multiple interdependent factors in accurately assessing and comparing photocatalytic efficiency, including the availability of redox-active molecules in solution, excitation spot properties (size, wavelength, and intensity), and the MoS_2_ platform characteristics (thickness, size, and defect concentration). Overall, this study presents a robust methodology for evaluating the photocatalytic properties of materials under realistic operating conditions, allowing simultaneous assessment of both reduction and oxidation reactions without measurement-induced artifacts.


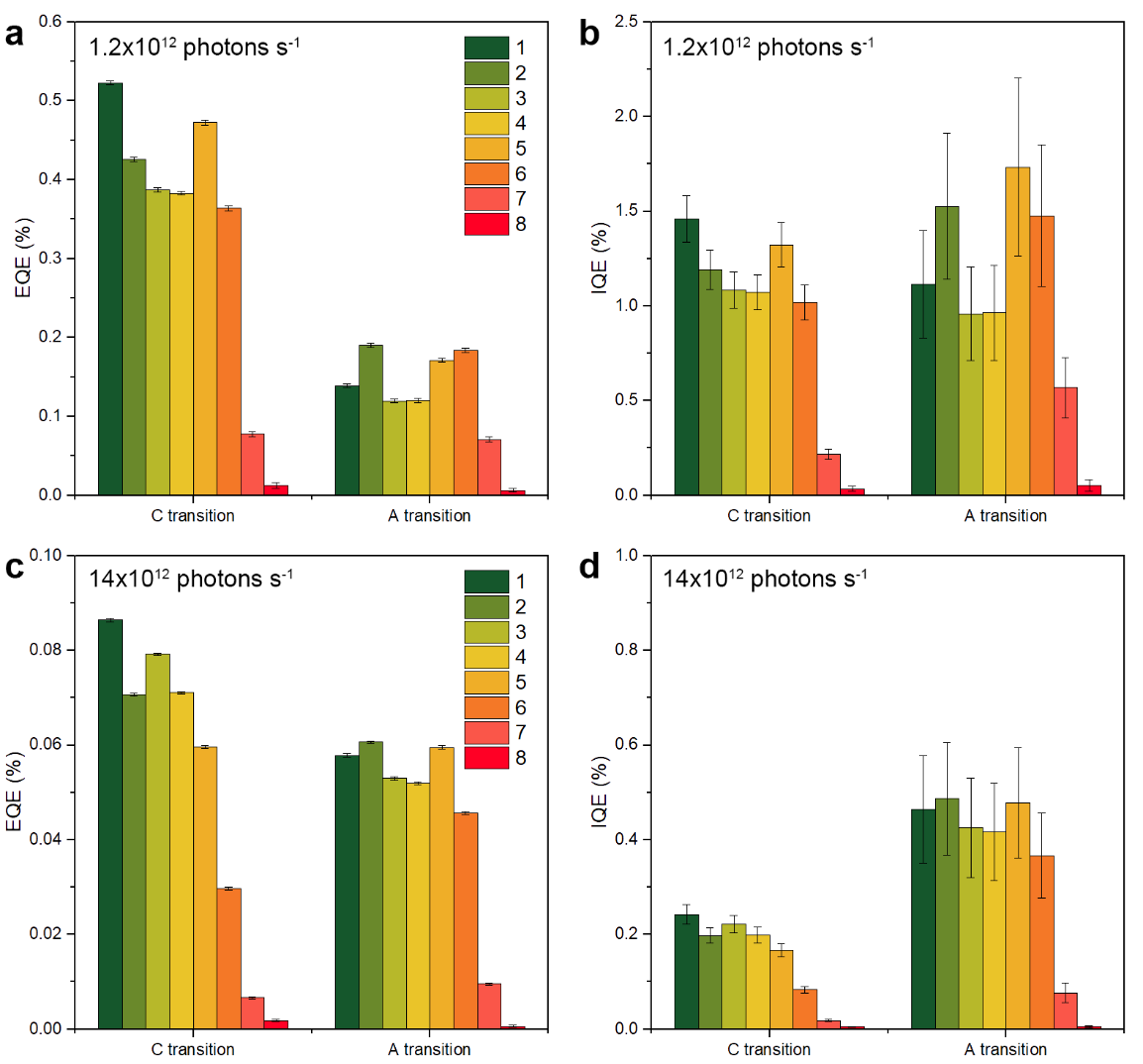


**Fig. S16:** **Quantum efficiency evolution according to the population of MoS_2_ flakes.** (**a,c**) External and (**b,d**) internal quantum efficiency of different MoS_2_ flakes (Fig. S15) according to the excited transition at (**a,b**) ~1.2$\times$10^12^ and (**c,d**) ~14$\times$10^12^ photons s^-1^ (C transition at 455 nm; A transition at 660 nm). The error bars represent the standard deviation. The number of the flakes was assigned from the biggest to the smallest flakes (see Fig. S15) with gradient colors from flake 1 (dark green) to flake 8 (red).

**Table S1:** **Parameters of the different light sources employed.** Values of the different power density and photon flux utilized during SPECM measurements. The pulse n° corresponds to the order of pulse in the photoactivity measurements during chronoamperometry such as in Fig. 3b and S3.

|  | **Transition C: 455 nm** | | **Exciton B: 595 nm** | | **Exciton A: 660 nm** | |
| --- | --- | --- | --- | --- | --- | --- |
| **Pulse n°** | Power density (W cm^-2^) | Photon flux (x10^12^ s^-1^) | Power density (W cm^-2^) | Photon flux (x10^12^ s^-1^) | Power density (W cm^-2^) | Photon flux (x10^12^ s^-1^) |
| 1 | 0.61 | 1.10 | 0.36 | 0.84 | 0.19 | 0.50 |
| 2 | 1.71 | 3.07 | 0.89 | 2.10 | 0.52 | 1.36 |
| 3 | 2.86 | 5.15 | 1.50 | 3.53 | 0.89 | 2.33 |
| 4 | 8.30 | 14.93 | 3.82 | 8.99 | 2.64 | 6.88 |
| 5 | 14.51 | 26.11 | 6.57 | 15.46 | 5.33 | 13.92 |
| 6 | 17.23 | 30.99 | 7.64 | 17.97 | 6.45 | 16.84 |

**Supplementary References**

1. Zhou, W. *et al.* Synthesis of Few-Layer MoS2 Nanosheet-Coated TiO2 Nanobelt Heterostructures for Enhanced Photocatalytic Activities. *Small* **9**, 140–147 (2013).

2. Ye, L., Wang, D. & Chen, S. Fabrication and Enhanced Photoelectrochemical Performance of MoS2/S-Doped g-C3N4 Heterojunction Film. *ACS Appl. Mater. Interfaces* **8**, 5280–5289 (2016).

3. Ansari, A. *et al.* Photo-oxidation of sulfite ions in the presence of some iron oxides. *Journal of Photochemistry and Photobiology A: Chemistry* **87**, 121–125 (1995).

4. Wang, S., Zhou, T., Pan, Z. & Trusler, J. P. M. Diffusion Coefficients of N2O and H2 in Water at Temperatures between 298.15 and 423.15 K with Pressures up to 30 MPa. *J. Chem. Eng. Data* **68**, 1313–1319 (2023).

5. Cornut, R. & Lefrou, C. A unified new analytical approximation for negative feedback currents with a microdisk SECM tip. *Journal of Electroanalytical Chemistry* **608**, 59–66 (2007).
